# Overexpression of microRNA-155 increases IL-21 mediated STAT3 signaling and IL-21 production in systemic lupus erythematosus

**DOI:** 10.1186/s13075-015-0660-z

**Published:** 2015-06-09

**Authors:** Tue Kruse Rasmussen, Thomas Andersen, Rasmus Otkjær Bak, Gloria Yiu, Christian Møller Sørensen, Kristian Stengaard-Pedersen, Jacob Giehm Mikkelsen, Paul Joseph Utz, Christian Kanstrup Holm, Bent Deleuran

**Affiliations:** Department of Biomedicine, Aarhus University, 8000 Aarhus C, Denmark; Department of Medicine, Division of Immunology and Rheumatology, Stanford School of Medicine, Stanford, CA USA; Department of Rheumatology, Aarhus University Hospital, 8000 Aarhus C, Denmark

## Abstract

**Introduction:**

Interleukin (IL)-21 is a key cytokine in autoimmune diseases such as systemic lupus erythematosus (SLE) by its regulation of autoantibody production and inflammatory responses. The objective of this study is to investigate the signaling capacity of IL-21 in T and B cells and assess its possible regulation by microRNA (miR)-155 and its target gene suppressor of cytokine signaling 1 (SOCS1) in SLE.

**Methods:**

The signaling capacity of IL-21 was quantified by stimulating peripheral blood mononuclear cells (PBMCs) with IL-21 and measuring phosphorylation of STAT3 (pSTAT3) in CD4+ T cells, B cells, and natural killer cells. Induction of miR-155 by IL-21 was investigated by stimulating purified CD4+ T cells with IL-21 and measuring miR-155 expression levels. The functional role of miR-155 was assessed by overexpressing miR-155 in PBMCs from SLE patients and healthy controls (HCs) and measuring its effects on STAT3 and IL-21 production in CD4+ and CD8+ T cells.

**Results:**

Induction of pSTAT3 in CD4+ T cells in response to IL-21 was significantly decreased in SLE patients compared to HCs (*p* < 0.0001). Further, expression levels of miR-155 were significantly decreased and SOCS1 correspondingly increased in CD4+ T cells from SLE patients. Finally, overexpression of miR-155 in CD4+ T cells increased STAT3 phosphorylation in response to IL-21 treatment (*p* < 0.01) and differentially increased IL-21 production in SLE patients compared to HCs (*p* < 0.01).

**Conclusion:**

We demonstrate that SLE patients have reduced IL-21 signaling capacity, decreased miR-155 levels, and increased SOCS1 levels compared to HCs. The reduced IL-21 signaling in SLE could be rescued by overexpression of miR-155, suggesting an important role for miR-155 in the reduced IL-21 signaling observed in SLE.

**Electronic supplementary material:**

The online version of this article (doi:10.1186/s13075-015-0660-z) contains supplementary material, which is available to authorized users.

## Introduction

Systemic lupus erythematosus (SLE) is an autoimmune disease characterized by inflammation in multiple organ systems [1]. A hallmark of SLE is the production of autoreactive antibodies directed at a conserved group of nuclear antigens [2, 3]. The cause of this loss of tolerance is not well described but presumably involves dysregulated cytokine signaling in T and B cells. In recent years follicular T helper (Tfh) cells have emerged as key players in controlling and promoting germinal center (GC) formation and B cell functions [4, 5]. Tfh cells are defined by their production of interleukin (IL)-21 and expression of a panel of surface markers including programmed death (PD)-1 and CXC chemokine receptor (CXCR)5 [4]. Previous studies have shown that IL-21 production is increased in CD4+ T cells from SLE patients [6]. A key non-redundant function of IL-21 in the context of autoimmunity is to act as a gatekeeper for B cell proliferation and differentiation into antibody-producing plasma cells [7–9]. Tfh cells signal to B cells directly via cell–cell contact as well as through secretion of cytokines like IL-21 [8]. In combination with the appropriate co-stimulatory signals, IL-21 is capable of inducing class-switching and B cell proliferation independent of antigen binding and B cell receptor (BCR) signaling [9]. Under physiological conditions Tfh cells control and limit GC formation in addition to B cell maturation. However, in conditions where Tfh cells and IL-21 are increased, elevated autoantibodies and IgG levels are observed in mice and humans [9–13].

IL-21 signals via the JAK/STAT signaling pathway and induces phosphorylation of STAT3 but also STAT1 and STAT5. Upon phosphorylation, STAT3 forms a homodimer, which translocates to the nucleus where it initiates transcription of target genes – including the *IL21* gene. This forms an autocrine loop for IL-21 production via STAT3 phosphorylation [14, 15]. The STAT signaling pathways are regulated at multiple levels, and the STAT-induced STAT inhibitors (SSI) form a key negative feedback loop for regulation and attenuation of STAT signals. Recently, it has been shown that a member of this family, suppressor of cytokine signaling 1 (SOCS1), negatively regulates STAT3 phosphorylation by preventing STAT3 from binding to JAK [16, 17].

MicroRNAs (miRNAs) are major regulators of gene expression by initiating degradation and inhibiting translation of mRNAs from target genes. Recent studies have suggested that regulation of immune responses by miRNAs play a role in SLE pathogenesis [18, 19]. MicroRNA-155 (miR-155) has many effects, but one of its best described roles is regulation of cell signaling in both T and B cells by targeting signaling repressors [20, 21]. Recently, miR-155 was shown to target SOCS1, a key regulator of STAT3 and the main signaling molecule for IL-21 [21].

As new treatment strategies in autoimmune diseases aim to inhibit signaling responses through the JAK-STAT pathway it is becoming increasingly important to understand the regulation of this process [22]. We hypothesize that IL-21-induced IL-21 expression in CD4+ T cells from SLE patients is regulated and attenuated by miR-155 and SOCS1. To investigate this we quantified the expression levels and induction of miR-155 by IL-21 and the dependency on phosphorylation of STAT3. Finally, we examined the effects of miR-155 overexpression in CD4+ T cells on STAT3 phosphorylation and IL-21 production in SLE patients.

## Methods

### Patients

All SLE patients (n = 14) met the American College of Rheumatology (ACR) updated 1997 SLE criteria [23]. Samples were collected at the outpatient clinic at the Department of Rheumatology, Aarhus University Hospital. The SLE Disease Activity Index (SLEDAI) [24] and the Systemic Lupus International Collaborative Clinics (SLICC)/ACR Damage Index [25] as well as other clinical and paraclinical parameters were recorded at sampling (Table 1). Patients receiving high-dose prednisone (>15 mg/day) or biologics such as belimumab (Benlysta) were excluded from the study.

**Table 1:** Clinical characteristics of systemic lupus erythematosus patients and age- and gender-matched healthy controls

Parameters	SLE patients	HCs
	(n = 14)	(n = 12)
Age (years)	41.0 (26.0–52.0)	43.0 (30.0–51.0)
Gender (% female)	100	100
Disease duration (years)	4.6 (2.2–11.4)	-
SLEDAI	4.0 (2.5–6.0)	-
SLICC	0.0 (0.0–1.0)	-
dsDNA (% ever positive)	79.0	-
dsDNA (IU/mL)	31.5 (10.8–396.8)	-
Clinical features (no. of patients)		
Nephritis	0	-
Arthritis	4	-
Rash	7	-
Serositis	2	-
Leukopenia	6	-
Oral ulcers	4	-
Vasculitis	1	-
Treatment (no. of patients)		
None	2	-
Hydroxychloroquine	6	-
Azathioprine	4	-
Cyclophosphamide	1	-
Mycophenolate mofetil	3	-
Prednisone	12	-

Values are shown as median (interquartile range) or as number where indicated. *dsDNA* double-stranded DNA, *HC* healthy control, *SLE* systemic lupus erythematosus, *SLEDAI* SLE Disease Activity Index, *SLICC* Systemic Lupus International Collaborating Clinics

### Ethics

The study was approved by the Regional Ethics Committee (VEK2004-800-2) and the Danish Data Protection Agency (2006-41-6098). All samples were obtained after informed consent and in accordance with the Declaration of Helsinki and the study was carried out in accordance with the principles of the International Conference on Harmonization guidelines for Good Clinical Practice (1996 revision).

### Intracellular phosphoSTAT3 flow cytometry

Cells were thawed and rested overnight at 37 °C and 5 % CO_2_ in RPMI-1640 with 1 % glutamine, 2 % streptomycin and penicillin, and 10 % FCS (cell culture media). The following day, peripheral blood mononuclear cells (PBMCs) were stimulated at 37 °C for 0, 5, 15, 30, 60, 90, 120, or 150 minutes with 25 ng/ml recombinant human (rhu)-IL-21 (provided by Novo Nordisk A/S, Maaloev, Denmark). Fifteen minutes prior to the end of the stimulation cells were stained with anti-CD4 APC (clone: MT310, Dako, Gloestrup, Denmark), anti-CD19 AlexaFluor488 (clone: HIB19, BD Biosciences, Albertslund, Denmark) and anti-CD56 PC-7 (clone: A51078, Beckman Coulter, Copenhagen, Denmark). The cells were fixed in a 2 % formaldehyde solution. Hereafter, samples were permeabilized in ice-cold 90 % methanol and stained with anti-tyrosine-phosphorylated STAT3 (pY705) PE (clone: 4/P-STAT3, BD Biosciences) or anti-serine-phosphorylated STAT3 (pS727) PE (clone: 49/p-Stat3, BD Biosciences) for 60 minutes at room temperature. Samples were analyzed within 4 hours. Data were analyzed using FlowJo 9.7.2 (Tree Star Inc., Ashland, Or, USA).

### Intracellular IL-21 flow cytometry

For intracellular IL-21 staining, cells were stimulated for 4 hours with 50 ng/mL phorbol 12-myristate 13-acetate (Sigma-Aldrich, St. Louis, MO, USA) and 1 μg/mL ionomycin (Sigma-Aldrich) in the presence of 10 μg/mL Brefeldin A (Sigma-Aldrich). Cells were then stained with anti-CD4 APC (clone: MT310, Dako), CD45RO ECD (clone: UCHL1) and anti-CD56 PC-7 (clone: A51078, Beckman Coulter). Cells were permeabilized using 0.3 % saponin in PBS/BSA/Azide. All samples were blocked with 10 % heat-inactivated normal mouse serum (in-house production) after permeabilization before being stained with anti-IL-21 PE antibody (clone: 3A3-N2, eBioscience, San Diego, CA, USA). All antibodies used were titrated to optimal working concentration. IL-21-positive cells were gated using a fluorescence minus-one control combined with an appropriate isotype matched for IgG subtype, concentration, species, and fluorochrome. A minimum of 50,000 events was recorded for all samples.

### Quantitative RT-PCR

RNA for mRNA and miRNA analyses was isolated using the High Pure RNA Isolation Kit (Roche, Hvidovre, Denmark) and miRNeasy mini kit (Qiagen, Venlo, Holland), respectively. The expression levels of IL-21 and SOCS1 were determined using predesigned primer and probe mixes (Applied Biosystems, Waltham, MA, USA) labeled with the FAM-BHQ system as fluorescence/quencher. Quantitative RT-PCR was performed using the TaqMan RNA-to-CT 1-Step Kit (Applied Biosystems) on a MX3005P RQ-PCR machine (Stratagene, La Jolla, CA, USA), using the following cycle parameters: 15 minutes at 48 °C, 10 minutes at 95 °C and 40 cycles 15 seconds at 95 °C and 1 minute at 60 °C. For normalization GAPDH, RPS11 and ACTB were used.

The expression levels of the mature miRNA hsa-mir-155-5p (hereafter referred to as miR-155) were determined using predesigned primer and probe mixes (Applied Biosystems). Complementary DNA was made using specific primers for each miRNA (as supplied in the kit) and the TaqMan MicroRNA Reverse Transcription kit (Applied Biosystems), using the following cycle parameters: 30 minutes at 16 °C, 30 minutes at 42 °C and 5 minutes at 85 °C followed by a RT-PCR reaction, using the following cycle parameters: 2 minutes at 50 °C, 10 minutes at 95 °C and 40 cycles 15 seconds at 95 °C and 1 minute at 60 °C. For normalization of miRNAs, RNU6B and RNU43 were used.

For all experiments, individual samples were run in duplicate with a maximum acceptable inter-replicate difference of 1 cycle. Data were calculated using the ∆Ct method and expressed relative to the geometric mean of the housekeeping genes.

### Cell culture and cell purification

In respective experiments CD4+ T cells from SLE patients and healthy controls (HCs) were cultured at 37 °C and 5 % CO_2_ in cell culture media and stimulated with 50 ng/mL of rhuIL-21 for 0, 1, 3, and 6 hours prior to RNA purification. While this dose is clearly supraphysiological, previous studies have found 25–100 ng/mL to be optimal doses to study both IL-21 signaling as well as induction of gene transcription [14, 15, 26]. CD4+ T cells were purified using EasySep Untouched CD4+ T cells (Stemcell, Vancouver, Canada). For selected experiments cells were treated with 30 μM cucurbitacin I (CucI) (Sigma Aldrich) to selectively inhibit phosphorylation of STAT3. As toxic effects of CucI on cells in culture could potentially confound our results we tested the effect of CucI on viability in cell cultures as well as the specificity of its inhibition of STAT3 phosphorylation (Additional file 1: Figure S1A-B).

### MicroRNA overexpression

Lentiviral vectors were produced as previously described [27]. Verification of efficient miRNA expression was performed by transfection of HEK293 cells with the lentiviral transfer plasmid, followed by quantification of miR-155 by RT-PCR (Additional file 2: Figure S2). The biological titer of the lentiviral vector preparations was determined using flow cytometric analysis of enhanced green fluorescent protein (eGFP) expression in HEK293 cells transduced with serially diluted vectors, and vector amounts were normalized according to the obtained titers. Cells were treated with lentiviral vectors encoding miR-155 + eGFP or eGFP only and PBS. Prior to transduction, PBMCs were stimulated with CD3 and CD28 for 24 hours in 37 °C and 5 % CO_2_ in cell culture media. Lentiviral vectors were then added directly to the cell culture and incubated for 24 hours. Subsequently, the cells were washed with PBS and cultured in cell culture media supplemented with 2 μg/mL IL-2 (Proleukin®, Chiron Emeryville, CA, USA) for 72 hours.

### Statistics

Statistical analyses were performed using GraphPad Prism 6.0d for Mac (GraphPad Software, La Jolla, CA, USA). Non-paired, non-parametric data were analyzed by Mann–Whitney *U* test. Correlation of non-parametric paired data was tested using Spearman’s Rho. Differences between datasets with multiple observations within each group were analyzed using a repeated measures (RM) two-way analysis of variance (ANOVA). In all tests the level of significance was a two-sided *p* value of less than 0.05. All values shown in text are median with interquartile range unless otherwise stated.

## Results

### Basal IL-21 production is increased in memory CD4+ T cells from SLE patients and provides possible feedback for IL-21 induction via STAT3

As IL-21 is implicated in SLE pathogenesis, we examined the main source of IL-21 in SLE. To further investigate this point we analyzed IL-21 production in naïve and memory CD4+ T cells as well as natural killer (NK) T cells from SLE patients and HCs. We also quantified the mRNA levels of IL-21 in CD4+ T cells from SLE patients and HCs and the capacity of IL-21 to induce IL-21 mRNA production by stimulating CD4+ T cells with IL-21.

**Fig. 1:**
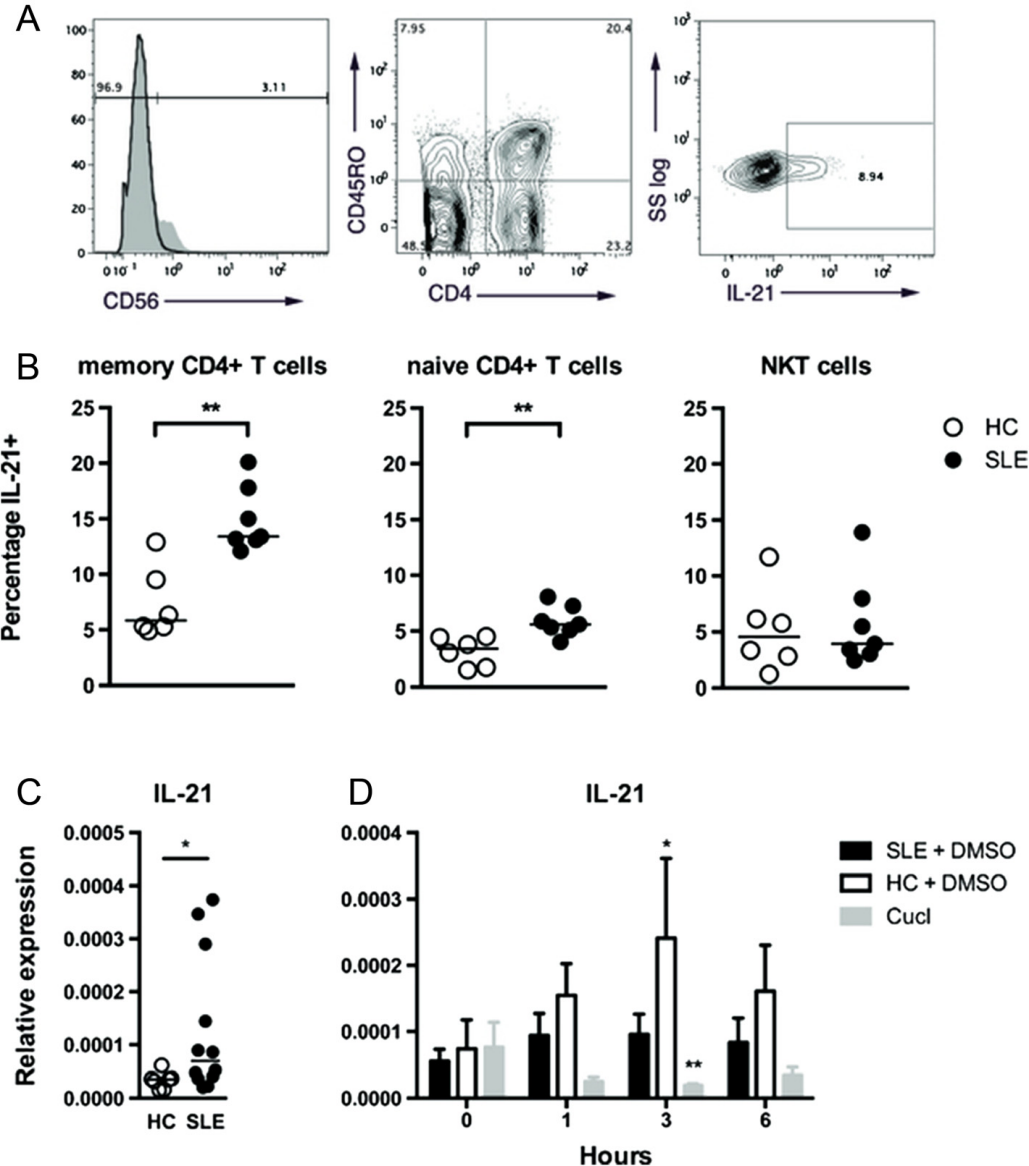
**a** Gating strategy for flow cytometry analyses of interleukin-21 (*IL-21*) production in CD4+CD45RO+/− T cells and CD4+CD56+ NK T cells. An fluorescence minus-one control was used to gate on CD56. **b** IL-21 production in immune cell subsets. Expression levels of IL-21 measured by flow cytometry in immune subsets of cells from systemic lupus erythematosus (*SLE*) patients (n = 6) and healthy controls (*HC*) (n = 7). Memory Th cells (CD4+CD45RO+) and naïve Th cells (CD4+CD45RO-) from SLE patients showed increased production of IL-21 upon stimulation compared to HC. Bar indicates median. **p < 0.01, ***p < 0.001 (Mann–Whitney *U*-test). **c** IL-21 mRNA expression levels. mRNA expression levels of IL-21 in purified CD4+ T cells from SLE patients (n = 12) and HC (n = 7). Bar indicates median. *p < 0.05 (Mann–Whitney *U*-test). **d** Autocrine induction of IL-21. mRNA expression levels of IL-21 in purified CD4+ T cells from SLE (n = 6) patients and HCs (n = 5) stimulated with IL-21 at indicated time points with and without the STAT3 inhibitor cucurbitatin I (*CucI*). Cells not treated with CucI were treated with the vehicle (*DMSO*). After 3 hours of IL-21 stimulation, IL-21 expression levels were significantly higher in DMSO-treated HC and significantly lower in CucI-treated CD4+ T cells compared to their respective baselines. Graph shows mean ± SEM. *p < 0.05 (Mann–Whitney *U*-test). *NK* natural killer, *SS* side-scatter

The percentage of memory CD4+CD45RO+ T cells producing IL-21 was significantly upregulated in SLE patients (13.4 %, 13.1–18.8 %) vs. HCs (5.8 %, 5.2–10.35 %; *p* < 0.001; Fig. 1b). Naïve CD4+CD45RO– T cells also showed increased IL-21 production but to a lesser degree as compared to HCs (5.6 %, 5.1–7.3 %, vs. 3.4, 1.7–4.4 %; *p* < 0.01). In CD4+CD56+ NK T cells, IL-21 levels were comparable to naïve CD4+ T cells although no difference was observed between SLE patients and HCs.

Supporting these findings, purified unstimulated CD4+ T cells from SLE patients had significantly increased IL-21 mRNA levels compared to HCs (*p* < 0.05; Fig. 1c). We found that the protein and mRNA levels of IL-21 in CD4+ T cells, as measured by flow cytometry and quantitative PCR respectively, correlated positively as tested by Spearman’s Rho although not significantly (*p* = 0.66, rho = 0.21). In contrast to recent studies we found no correlation between IL-21 mRNA levels and SLEDAI (Additional file 3: Figure S3) [28].

When analyzing IL-21 induction of IL-21 transcription in purified CD4+ T cells from SLE patients and HCs, we found that after 3 hours of IL-21 stimulation IL-21 mRNA levels were significantly increased in HCs but not in SLE patients (Fig. 1d). This induction was dependent on phosphorylation of STAT3, the main IL-21 signaling molecule, as inhibition of STAT3 phosphorylation with CucI blocked the response (Fig. 1d).

### IL-21-induced STAT3 phosphorylation is depressed in T and B cells from SLE lymphocytes

Ligation of IL-21R with IL-21 leads to STAT3 phosphorylation. Previously, mutations in the IL-21 receptor and dysregulation of STAT signaling have been linked to SLE pathology [29]. STAT3 has two separate phosphorylation sites (Y705 and S727) of which cytokine stimulation preferentially induces Y705, whereas S727 is more influenced by the basal activation state of the cell [30]. Here, we measured phosphorylation at both sites in CD4+ T cells, NK cells, and B cells stimulated with IL-21 for the indicated time periods.

**Fig. 2:**
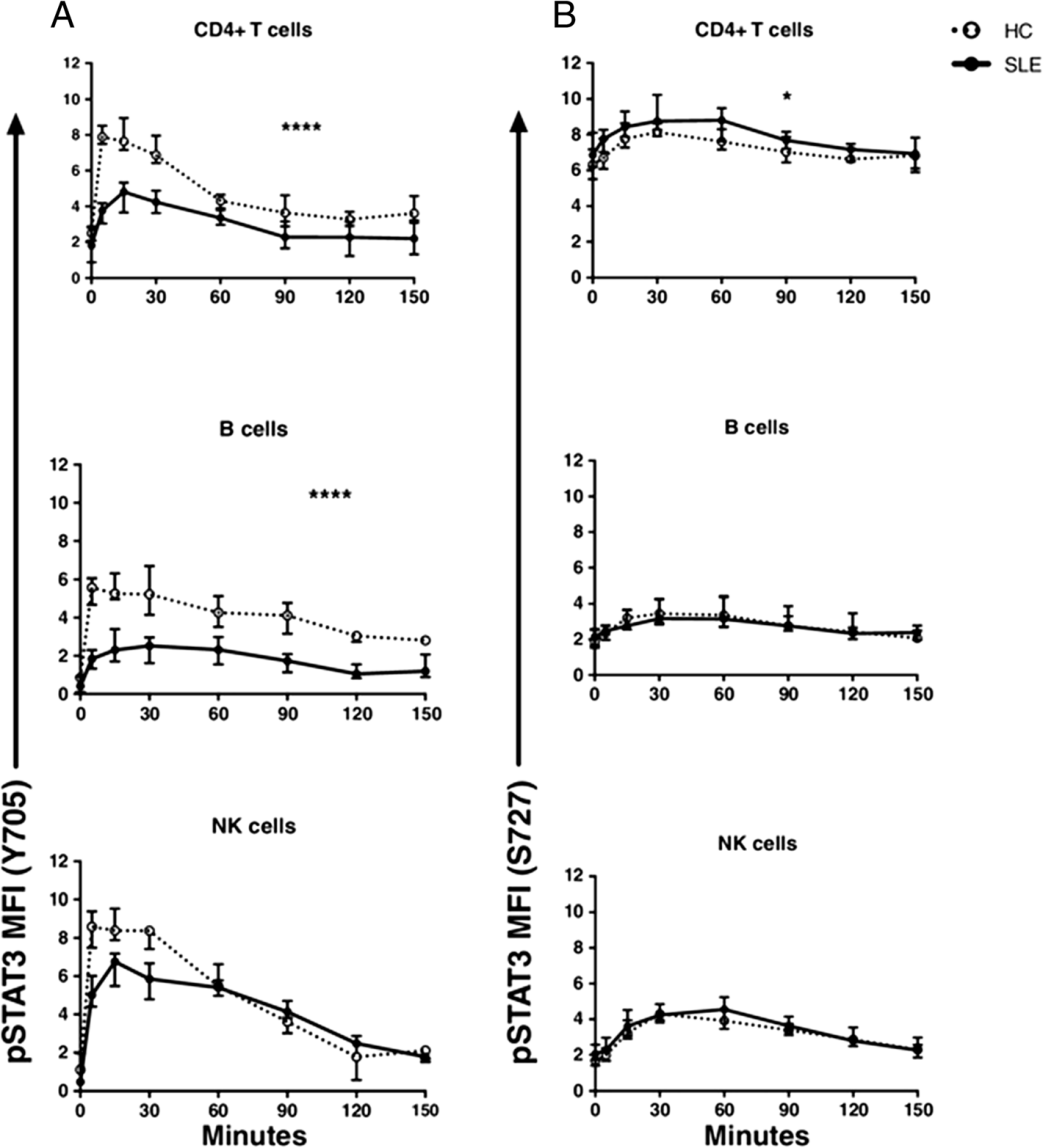
**a, b** IL-21 induced STAT3 phosphorylation. PBMCs from systemic lupus erythematosus (*SLE*) patients (*black line*) and healthy controls (*HC*) (*dotted line*) were stimulated with IL-21, and levels of phosphorylated STAT3 (*pSTAT3*) at tyrosine 705 (*Y705*) (**a**) and serine 727 (*S727*) (**b**) was measured in CD4+ T cells, B cells, and natural killer (*NK*) cells from SLE patients and HC (both n = 8). Graph shows median fluorescence intensity (*MFI*) with interquartile range. *p < 0.05, ****p < 0.0001 (RM two-way ANOVA)

The time-dependent stimulation showed a marked increase in STAT3 phosphorylation both in SLE patients and HCs upon IL-21 stimulation. The phosphorylation of STAT3 peaked between 5 and 15 minutes for the Y705 site and between 30 and 60 minutes for the S727 site (Fig. 2a, b). The induction of STAT3 by IL-21 resulted in a 3- to 10-fold increase in median fluorescence intensity (MFI). However, STAT3 phosphorylation was significantly lower in CD4+ T and B cells from SLE patients at the Y705 site (all *p* < 0.0001) compared to HCs. Conversely, phosphorylation of S727 was marginally but significantly increased in T cells (*p* < 0.05), but not in B or NK cells. Baseline levels of p-S727-STAT3 were not significantly increased in SLE patients compared to HCs.

### MicroRNA-155 expression is decreased and not inducible by IL-21 in SLE patients

miRNAs are ubiquitous regulators of gene expression and function mainly by facilitating the degradation of mRNAs harboring partially complementary target sites [31]. We have demonstrated that STAT3 signaling is decreased in SLE patients compared to HCs and we speculated that miR-155 could in part be responsible for this effect. We therefore examined expression levels of miR-155 in CD4+ T cells from SLE patients at baseline and after IL-21 stimulation.

**Fig. 3:**
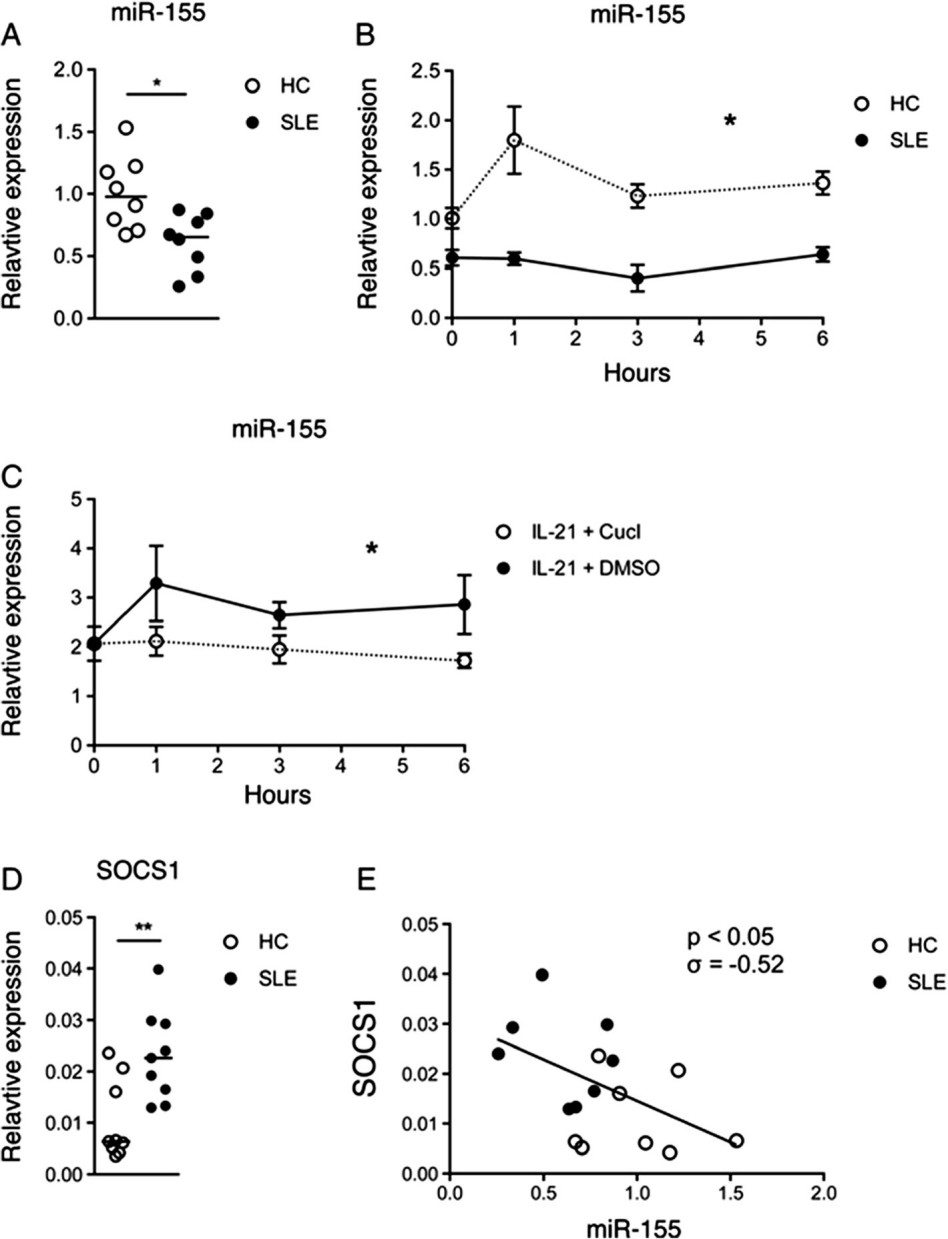
**a** Expression levels of microRNA-155 (*miR-155*) in CD4+ T cells. MiR-155 levels are significantly decreased in CD4+ T cells from systemic lupus erythematosus (*SLE*) patients (n = 7) compared with healthy controls (*HC*) (n = 8). Bar indicates median. *p < 0.05 (Mann–Whitney *U*-test). **b** Induction of miR-155 by IL-21. CD4+ T cells from SLE patients and HCs (both n = 3) were stimulated with IL-21 and expression levels of miR-155 were measured. Graph shows mean and ± SEM. *p < 0.05 (RM two-way ANOVA). **c** IL-21 mediated miR-155 induction is STAT3-dependent. Expression levels of miR-155 in CD4+ T cells upon IL-21 stimulation with either cucurbitacin I (*CucI*) or vehicle (*DMSO*) as control (both n = 3). Induction of miR-155 was significantly reduced by inhibiting STAT3 phosphorylation with CucI. Graph shows mean and ± SEM. *p < 0.05 (RM two-way ANOVA). **d** Expression levels of suppressor of cytokine signaling 1 (*SOCS1*) in SLE patients and HCs. SOCS1 expression levels are significantly increased in CD4+ T cells from SLE patients compared with HCs (both n = 9). Bar indicates median. **p < 0.01 (Mann–Whitney *U*-test). **e** Linear regression of expression levels of miR-155 versus SOCS1. The expression levels of miR-155 versus SOCS1 in CD4+ T cells correlated negatively and significantly (Spearman’s rank correlation) in SLE patients (n = 8) compared to HCs (n = 8)

At baseline, miR-155 was significantly decreased in SLE patients versus HCs (Fig. 3a). Following IL-21 stimulation, increased miR-155 levels peaked in HCs at 1 hour and remained above baseline levels throughout the time course (Fig. 3b). At no time point did miR-155 in SLE patients show increased levels as compared to the baseline levels. This difference in induction levels was significant, as evaluated by a RM two-way ANOVA (*p* < 0.05). Relative expression levels of miR-155 showed no correlation to SLEDAI or other clinical parameters.

As the observed effects on miR-155 could be attributed to alternate signal transduction independent of STAT3, we addressed this possibility by inhibiting STAT3 phosphorylation using CucI. PBMCs were stimulated with IL-21 for the time points indicated and phosphorylation of Y705 was measured by flow cytometry.

CD4+ T cells from HCs were stimulated with IL-21 with and without CucI and miR-155 levels were measured. Induction of miR-155 by IL-21 was significantly inhibited with CucI treatment (*p* = 0.002) (Fig. 3c). Combined, these findings support that the effects observed on miR-155 are mediated through the activities of IL-21 and STAT3.

### Expression of SOCS1 is increased in CD4+ T cells from SLE patients

Recently, it has been shown that miR-155 inhibits SOCS1 function [21]. The SOCS family of proteins is known to negatively regulate signaling of the IL-2 cytokine family members by interfering with JAK binding and STAT phosphorylation. Previous work has also suggested that SOCS1 specifically inhibits IL-2 family cytokines, like IL-21, which signal via the common gamma chain [16].

CD4+ T cells from SLE patients exhibited significantly increased levels of SOCS1 compared to HCs (Fig. 3d). Further, expression levels of miR-155 in CD4+ T cells from SLE patients and HCs combined correlated negatively with expression levels of SOCS1 (Fig. 3e).

### Overexpression of miR-155 increases STAT3 phosphorylation by IL-21 in CD4+ T cells

We have shown that CD4+ T cells from SLE patients have low levels of miR-155 and high levels of SOCS1, which in turn inhibits STAT3 phosphorylation in SLE patients. We speculate that this link functions as a negative feedback mechanism for IL-21 production as an attenuation of the high levels of IL-21 produced by CD4+ T cells in SLE patients. To confirm the effects of miR-155 on STAT3 phosphorylation and IL-21 production, we overexpressed miR-155 using a lentiviral vector system. In brief, PBMC from SLE patients and HCs (both n = 10) were transduced with either a vector containing the GFP gene only or a vector expressing both eGFP and miR-155. After transduction, cells were allowed to rest for 72 hours to obtain optimal levels of miR-155 production. The cells were subsequently stimulated with IL-21 for the time points indicated and stained for phosphorylated STAT3 (pSTAT3; Y705) as described above.

**Fig. 4:**
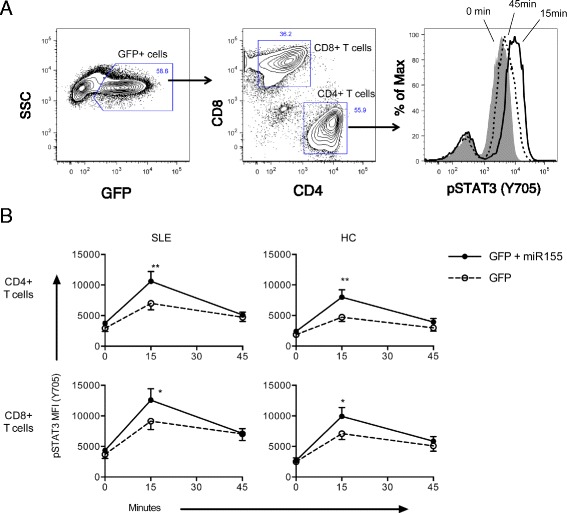
**a** Gating strategy used to quantify STAT3 phosphorylation in transduced cells. In brief, transduced cells were gated as green fluorescent protein (*GFP*) + and within this population CD4+ and CD8+ T cells were gated and mean fluorescence intensities (*MFI*) were measured for phosphorylated STAT3 (*pSTAT3*; *Y705*) at 0, 15, and 45 minutes after IL-21 stimulation. **b** IL-21 induced STAT3 phosphorylation after miR-155 transduction. MFI of pSTAT3 (Y705) upon IL-21 stimulation in transduced cells from systemic lupus erythematosus (*SLE*) patients (n = 10) and healthy controls (*HC*) (n = 10) gated on CD4+ and CD8+ T cells. Phosphorylation of STAT3 was significantly higher after 15 minutes of IL-21 stimulation in both CD4+ and CD8+ T cells from SLE patients and HCs, but not at baseline or after 45 minutes. In brief, PBMCs were transduced using lentiviral vectors carrying either the GFP gene alone or genes encoding GFP and miR-155. *p < 0.05, **p < 0.01 (Mann–Whitney *U*-test). *SSC* side-scatter

Overexpression of miR-155 resulted in significantly increased levels of STAT3 phosphorylation in both CD4+ and CD8+ T cells from SLE patients and HCs (Fig. 4a, b). For all subsets, the increase was most pronounced after 15 minutes of IL-21 stimulation with only marginal upregulation after 45 minutes. The effect of overexpressing miR-155 was consistent across SLE patients and HCs as no differences in STAT3 phosphorylation were observed when comparing either CD4+ or CD8+ T cells from SLE patients with cells derived from HCs.

### IL-21 production increases upon miR-155 overexpression in CD4+ T cells from SLE patients

To address the impact of the observed miR-155 downregulation and its impact on IL-21 production in SLE patients, cells transduced with the miR-155-encoding vector were stimulated as described above and IL-21 production capacity was measured.

**Fig. 5:**
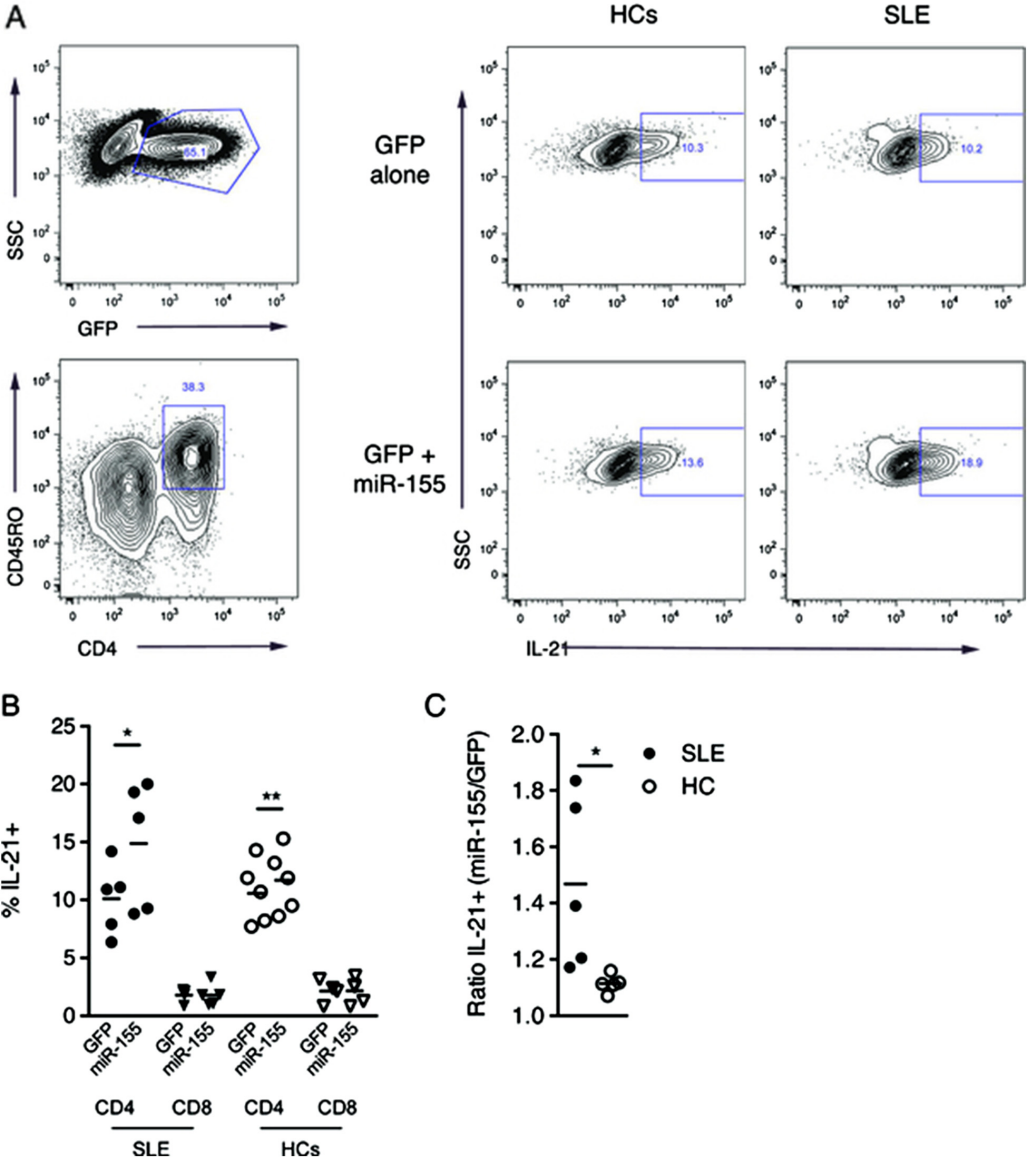
**a** Gating strategy used to quantify interleukin-21 (*IL-21*) production capacity after lentiviral transduction. Transduced cells were gated as green fluorescent protein (*eGFP*) + and within this population CD4+CD45RO+ T cells were selected and the percentages of IL-21+ cells were measured. Panel shows healthy controls (*HCs*) and systemic lupus erythematosus (*SLE*) patients transduced with either GFP alone or GFP + microRNA-155 (*miR-155*) (both n = 5). No CD45RO– populations were observed after the transduction and subsequent incubation. **b** Percentages of IL-21 CD4+CD45RO+ and CD8+CD45RO+ T cells from SLE patients and HCs transduced with either miR-155 or GFP alone. CD4+ T cells transduced with miR-155 from both SLE patients and HCs showed a significant increase in IL-21 production compared to GFP controls. *p < 0.05, **p < 0.01. **c** Ratio of IL-21 (miR-155/eGFP) expression upon miR-155 transduction. CD4+CD45RO+ T cells from SLE patients had significantly higher IL-21 upregulation compared with HCs (ratios 1.47 vs. 1.09, respectively; *p* < 0.05). CD8+CD45RO+ T cells are low level producers of IL-21 and were included as controls. No significant difference in upregulation of IL-21 production between SLE patients and HCs was seen in CD8+CD45RO+ T cells. *p < 0.05. *SSC* side-scatter

IL-21 production was significantly increased in CD4+ T cells transduced with miR-155 compared to GFP controls from both SLE patients and HCs as measured by the percentage of IL-21 positive cells (Fig. 5b). CD8+ T cells are low level producers of IL-21 and were included as controls. IL-21 production in CD8+ T cells from HCs and SLE patients was not significantly upregulated when comparing miR-155 transduced to GFP controls. Comparing the ratio of IL-21-positive cells in miR-155-transduced versus GFP-control SLE patients showed a significantly higher ratio of IL-21 production increase (Fig. 5c).

## Discussion

In this study we demonstrate a functional role for miR-155 in regulating STAT3 phosphorylation and IL-21 production in CD4+ T cells from SLE patients. We show that SLE patients have decreased STAT3 phosphorylation upon IL-21 stimulation and that SLE patients have low levels of miR-155 and correspondingly high levels of SOCS1 compared to HCs. While overexpression of miR-155 leads to higher levels of STAT3 phosphorylation both in SLE patients and HCs, resulting IL-21 production is increased in SLE patients compared to HCs.

Several studies have investigated the role of IL-21 in SLE and its possible impact on SLE pathology. Recently, Dolff et al. [6] found that IL-21 production was increased in CD4+ but not CD8+ T cells from SLE patients compared to HCs. Interestingly, this study found no difference between inactive (SLEDAI <4) and active (SLEDAI >4) SLE patients and IL-21 production did not correlate to SLEDAI scores. This indicates that high IL-21 production is a robust feature of SLE pathology and is seemingly not affected by disease activity or treatment. Confirming and expanding on these findings we found similarly high IL-21 levels in CD4+ T cells but further demonstrate that the principal producers of IL-21 are found in the memory compartment of CD4+CD45RO+ T cells. These findings are in line with other studies showing that naïve T cells require combinations of T cell receptor stimuli and cytokines such as IL-6 and transforming growth factor beta to become potent producers of IL-21 [32, 33].

While other studies have shown that SLE patient with high SLEDAI (active SLE) had increased IL-21 mRNA levels we were not able to recapitulate these findings [28]. However, as our data show there is an apparent subsetting of IL-21 mRNA levels and production in the SLE patients. This apparent subsetting did not reflect a specific treatment regime such as high-dose prednisone. An alternative explanation for the subsetting could be found in the interferon signature as initially published by Baechler et al. [34]. IL-21 is indeed part of the interferon signatures and would thus in part reflect this in our data. Further, while the interferon signature predicts severe complications such as nephritis and involvement of the central nervous system it does not directly correlate with SLEDAI [34].

Two studies by Caprioli et al. and Nurieva et al. have demonstrated that IL-21 is capable of functioning in an autocrine manner in human T cells by inducing its own production [14, 15]. This autocrine induction is dependent upon binding of STAT3 to the IL-21 promoter region, as ablation and inhibition of STAT3 inhibits IL-21 production [14, 15]. We found increased IL-21 mRNA expression in SLE patients versus HCs and that IL-21 was capable of inducing IL-21 mRNA expression in a STAT3-dependent manner both in SLE patients and HCs, although no difference in induction was observed. Interestingly, inhibition of STAT3 phosphorylation tended to decrease IL-21 mRNA levels below levels observed in untreated cells. This presumably reflects inhibition of baseline pSTAT3 induction of IL-21 mRNA transcription.

STAT phosphorylation is an area of increasing importance in SLE, especially with the introduction of the JAK/STAT inhibitors as new treatment options in autoimmune disease [22]. IL-21 signals almost exclusively through STAT phosphorylation, mainly STAT3 but also STAT1 and STAT5 [35]. Only a few studies exist examining the intracellular signaling network in SLE and how it could be “re-wired” by tonic stimulation with immunoregulatory cytokines such as IL-21. In our study, we demonstrate that while IL-21 production is increased in SLE there is a marked suppression of STAT3 phosphorylation upon IL-21 stimulation of CD4+ T cells. Further, expression of a key inhibitor of STAT3 phosphorylation, SOCS1, is also correspondingly increased. We interpret the downregulation of IL-21 signaling, mediating key pathological signals both in T and B cells, as an attenuation of previously increased levels of STAT3 phosphorylation due to increased and tonic IL-21 stimulation. In support of this and in line with our findings, Huang et al*.* found that baseline STAT3/5 phosphorylation correlated positively with disease activity in SLE patients as measured by SLEDAI, and that STAT3 phosphorylation was significantly depressed in T and B cells from SLE patients in response to IFNα and IL-6 [36]. Additional studies by Hale et al. [17] led to similar findings in a spontaneous murine model of SLE, the MRL/*lpr* mouse. IL-21-mediated STAT3 phosphorylation was increased early in the disease course and downregulated as disease progressed until levels decreased below those of healthy control MRL/++ mice. Expanding on this finding the authors demonstrated that this attenuation of increased STAT signaling early in disease was inversely correlated with upregulation of SOCS1 [17].

MicroRNAs have recently been suggested as important regulators of immune processes in SLE [19, 37, 38]. MiR-155 was recently shown to repress SOCS1 expression, and overexpression of miR-155 led to decreased levels of SOCS1 and constitutive activation of STAT3 [21]. Further, it has been shown that not only does miR-155 regulate STAT3 via SOCS1, it is also induced by STAT3 [39]. Studies of the role of miR-155 in experimental autoimmune encephalitis (EAE) by O’Connell et al*.* [40] found that mice deficient of the *miR155* gene were highly resistant to EAE due to inhibition of CD4+ T cell-mediated inflammation in EAE. Reports on miR-155 in SLE are very limited, but Stagakis et al*.* [41] found increased miR-155 expression in CD4+ T cells from SLE patients (active and inactive combined) versus HCs although they did not comment on how miR-155 levels in inactive SLE patients compare to HCs. Our cohort of SLE patients have median SLEDAI of 3 (with an interquartile range of 0–5) so they largely represent the inactive SLE cohort as defined by Stagakis et al*.* Mirroring our findings, Thai et al. found that ablation of miR-155 reduced disease activity and autoantibody levels in MRL/lpr mice [20]. These three above-mentioned studies combined indicate that parts of the T cell-driven autoimmune inflammatory process could be controlled by the levels of miR-155 [20, 40, 41].

Overall, these findings combined with our own support the idea that, early in disease, high levels of Th cell-specific cytokines such as IL-21 modulate the immune system and subvert its ability to maintain self-tolerance, ultimately leading to autoimmune disease such as SLE. To expand on this, we show that overexpression of miR-155 not only upregulates STAT3 responsiveness in CD4+ T cells, but also increases IL-21 production substantially more in CD4+ T cells from SLE patients compared to HCs. This differential upregulation of IL-21 production could indicate that it has been suppressed from high levels of production (potentially due to tonic cytokine stimuli and high levels of STAT activity) to a lower level – although still elevated compared to HCs.

## Conclusion

In conclusion, we demonstrate that IL-21 has reduced capacity to phosphorylate STAT3 and induce expression of IL-21 and miR-155 in CD4+ T cells from SLE patients. SLE patients also have correspondingly high levels of SOCS1, a documented target for miR-155. Further, overexpression of miR-155 increases STAT3 responsiveness and differentially increases IL-21 production in CD4+ T cells from SLE patients versus HCs. We suggest that this feedback loop exists to attenuate pathologically increased IL-21 production in CD4+ T cells from SLE patients.

## Additional files

Additional file 1: Figure S1. (A) To test whether or not CucI had apoptotic effects on CD4+ T cells PBMCs were incubated with either CucI or the carrier DMSO for two (gray shade) and eight hours (full line). Viability was assessed using Live/Dead viability stain. (B) Specificity of CucI STAT inhibition was assessed by flow cytometry by stimulating cells with IL-21 in the presence of either CucI or DMSO. Phosphorylation of STAT1/3/4/5 was assessed in both CD4+ and CD8+ T cells as well as B cells. Additional file 2: Figure S2. Verification of efficient miRNA expression was performed by transfection of HEK293 cells with the lentiviral transfer plasmid, followed by quantification of miR-155 by RT-PCR. Additional file 3: Figure S3. Linear regression of IL-21 mRNA levels in CD4+ T cells compared to SLEDAI. A Spearman Rank correlation was also performed (shown top right).

## Abbreviations

ACR: American College of Rheumatology
ANOVA: Analysis of variance
BCR: B cell receptor
CucI: Cucurbitacin I
CXCR: CXC chemokine receptor
EAE: Experimental autoimmune encephalitis
eGFP: Enhanced green fluorescent protein
GC: Germinal center
GFP: Green fluorescent protein
HC: Healthy control
IL: Interleukin
JAK: Janus kinase
MFI: Median fluorescence intensity
miRNA: MicroRNA
NK: Natural killer
PBMC: Peripheral blood mononuclear cell
PD: Programmed death
pSTAT3: Phosphorylated STAT3
RM: Repeated measures
rhu: Recombinant human
SLE: Systemic lupus erythematosus
SLEDAI: SLE Disease Activity Index
SLICC: Systemic Lupus International Collaborating Clinics
SOCS: Suppressor of cytokine signaling
SSI: STAT-induced STAT inhibitors
STAT: Signal transducer and activator of transcription
Tfh: Follicular T helper
