# Supplementary figures and images for: Overexpression of microRNA-155 increases IL-21 mediated STAT3 signaling and IL-21 production in systemic lupus erythematosus
Source: Arthritis Res Ther. 2015 Jun 9;17(1):154. doi: 10.1186/s13075-015-0660-z (PMC4504038; doi:10.1186/s13075-015-0660-z)

A

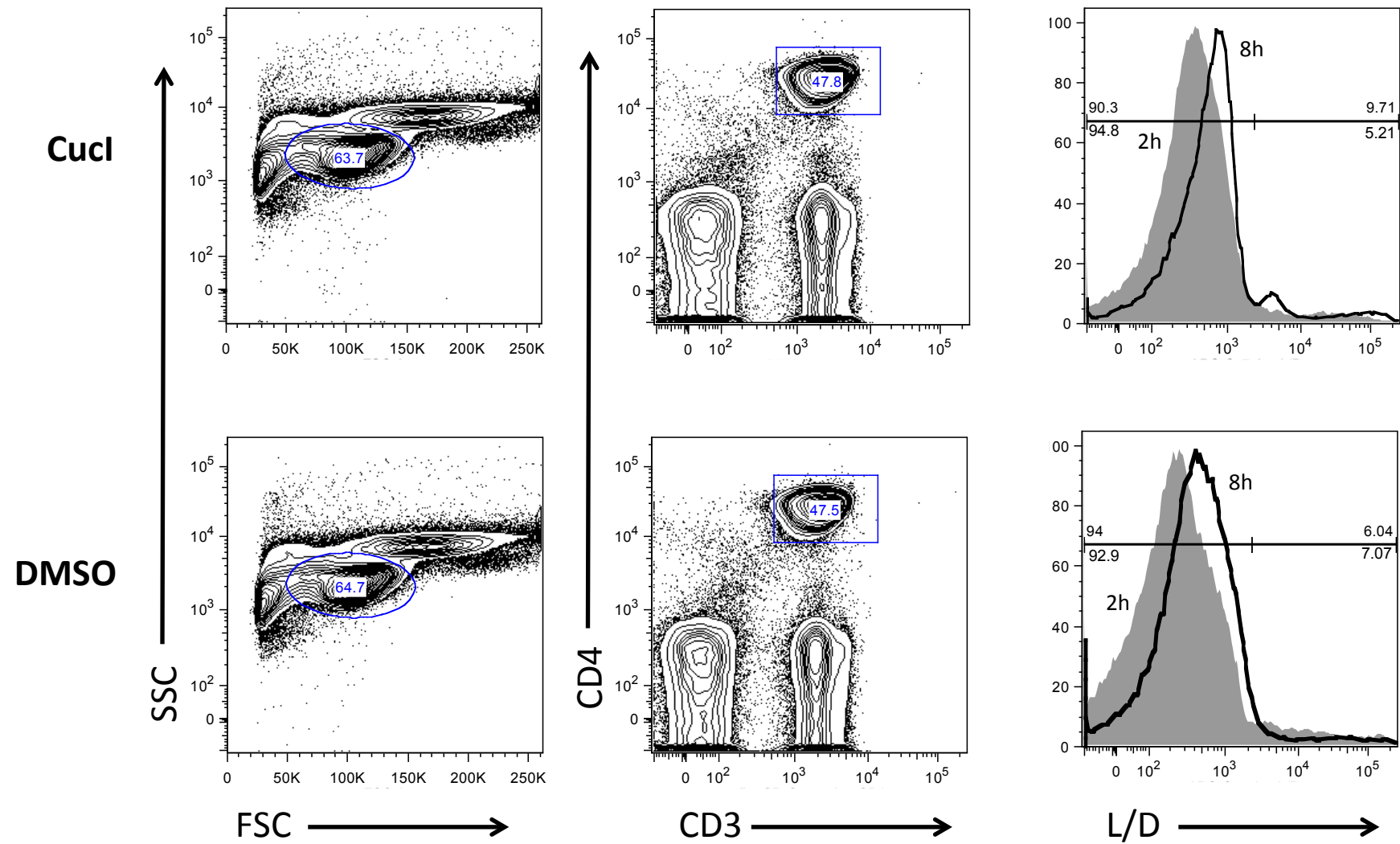

Supplement: Additional file 1: Figure S1. — (A) To test whether or not CucI had apoptotic effects on CD4+ T cells PBMCs were incubated with either CucI or the carrier DMSO for two (gray shade) and eight hours (full line). Viability was assessed using Live/Dead viability stain. (B) Specificity of CucI STAT inhibition was assessed by flow cytometry by stimulating cells with IL-21 in the presence of either CucI or DMSO. Phosphorylation of STAT1/3/4/5 was assessed in both CD4+ and CD8+ T cells as well as B cells. [file 13075_2015_660_MOESM1_ESM.zip › 13075_2015_660_MOESM1a_EM.pdf]

B

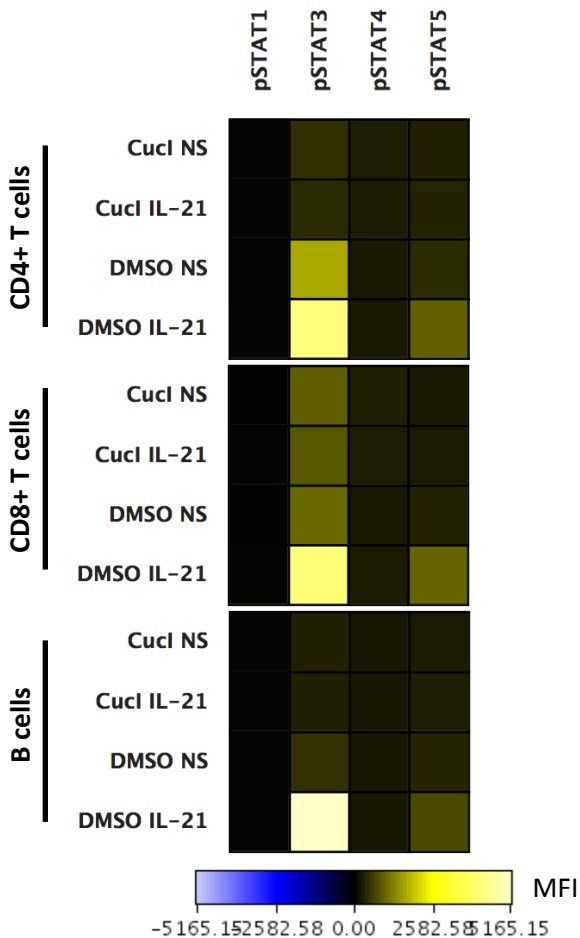

Supplement: Additional file 1: Figure S1. — (A) To test whether or not CucI had apoptotic effects on CD4+ T cells PBMCs were incubated with either CucI or the carrier DMSO for two (gray shade) and eight hours (full line). Viability was assessed using Live/Dead viability stain. (B) Specificity of CucI STAT inhibition was assessed by flow cytometry by stimulating cells with IL-21 in the presence of either CucI or DMSO. Phosphorylation of STAT1/3/4/5 was assessed in both CD4+ and CD8+ T cells as well as B cells. [file 13075_2015_660_MOESM1_ESM.zip › 13075_2015_660_MOESM1b_EM.pdf]

# miR-155

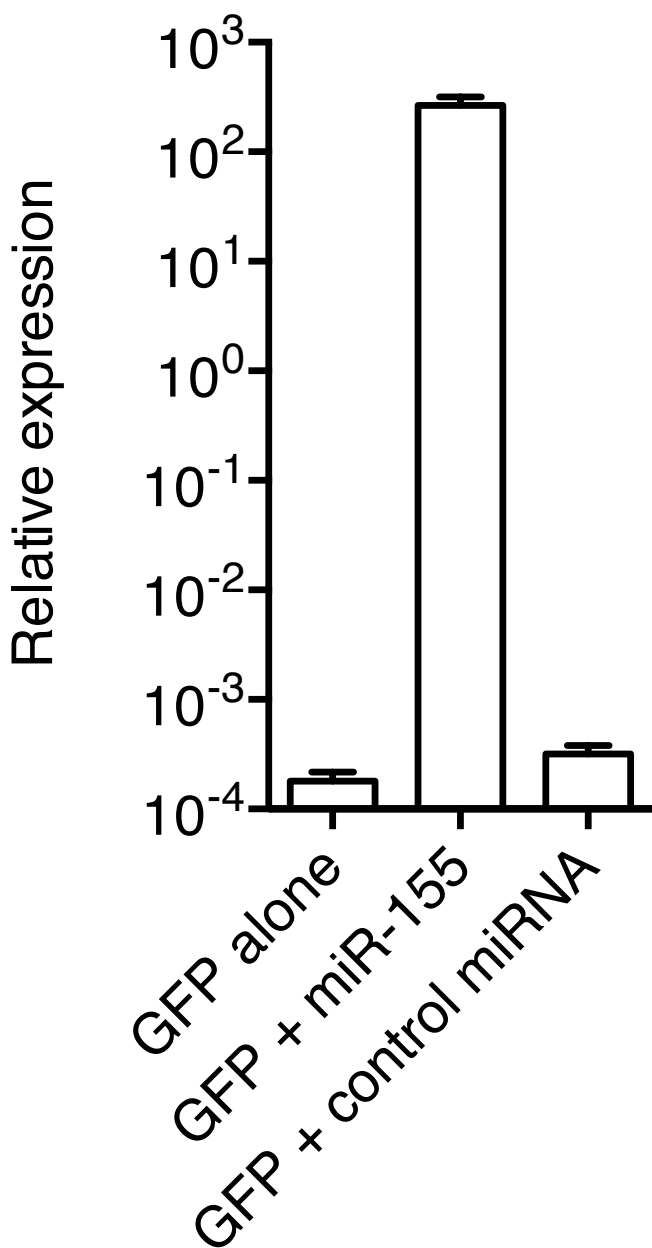

Supplement: Additional file 2: Figure S2. — Verification of efficient miRNA expression was performed by transfection of HEK293 cells with the lentiviral transfer plasmid, followed by quantification of miR-155 by RT-PCR. [file 13075_2015_660_MOESM2_ESM.pdf]

# IL-21 vs. SLEDAI

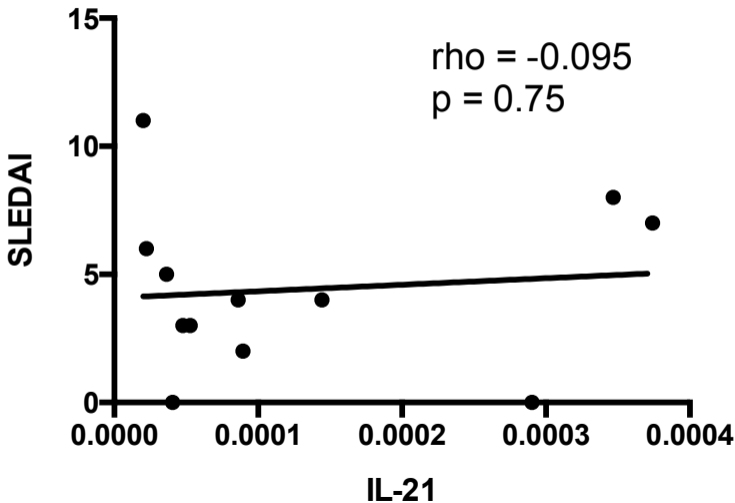

Supplement: Additional file 3: Figure S3. — Linear regression of IL-21 mRNA levels in CD4+ T cells compared to SLEDAI. A Spearman Rank correlation was also performed (shown top right). [file 13075_2015_660_MOESM3_ESM.pdf]
